# Supplementary material for: Polyketide synthase gene clusters in the ericoid mycorrhizal fungus Oidiodendron maius: genome-wide prediction, expression profiling, and functional characterization of knockout mutants
Source: Front Plant Sci. 2026 Sep 16;17:1924430. doi: 10.3389/fpls.2026.1924430 (PMC13623903; doi:10.3389/fpls.2026.1924430)
Supplement: Supplementary file 1 [file SupplementaryFile1.zip › Supplementary Materials/Supplementary Folder 1.zip!/Oidma1_AssemblyScaffolds_Repeatmasked/knownclusterblast/cluster57/jgi.p_Oidma1_107060_mibig_hits.html]

| MIBiG Protein | Description | MIBiG Cluster | MiBiG Product | % ID | % Coverage | BLAST Score | E-value |
| --- | --- | --- | --- | --- | --- | --- | --- |
| AGC45618.1 | non-ribosomal\_peptide\_synthetase | BGC0001394 | Phenalamide\_biosynthetic\_gene\_cluster | 34 | 79.2598303778 | 477 | 6.9e-134 |
| AAK57184.1 | MxaA | BGC0001022 | Myxalamid\_biosynthetic\_gene\_cluster | 34 | 80.0308404009 | 474 | 4.5e-133 |
| AAO23334.1 | NcpB | BGC0000397 | Nostocyclopeptide\_biosynthetic\_gene\_cluster | 33 | 75.0963762529 | 433 | 8.8e-121 |
| AHJ31215.1 | Long-chain-fatty-acid--CoA\_ligase | BGC0000430 | Spumigin\_biosynthetic\_gene\_cluster | 32 | 81.033153431 | 426 | 1.1e-118 |
| BAP27942.1 | nonribosomal\_peptide\_synthetase | BGC0001085 | Teleocidin\_B\_biosynthetic\_gene\_cluster | 32 | 81.8041634541 | 413 | 9.4e-115 |
| AFO85453.1 | non-ribosomal\_peptide\_synthetase | BGC0000391 | Methylpendolmycin/pendolmycin\_biosynthetic\_gene\_cluster | 33 | 80.1850424056 | 409 | 1.8e-113 |
| AGD80618.1 | non-ribosomal\_peptide\_synthetase | BGC0000394 | Naphthyridinomycin\_biosynthetic\_gene\_cluster | 32 | 72.8604471858 | 399 | 1.1e-110 |
| ACN39015.1 | putative\_nonribosomal\_peptide\_synthetase\_TomB | BGC0000448 | Tomaymycin\_biosynthetic\_gene\_cluster | 31 | 79.0285273709 | 396 | 9.1e-110 |
| ABI22133.1 | putative\_non-ribosomal\_peptide\_synthetase | BGC0000422 | Saframycin\_A\_biosynthetic\_gene\_cluster | 32 | 73.1688511951 | 388 | 4.2e-107 |
| EOY45602.1 | non-ribosomal\_peptide\_synthetase | BGC0001168 | Livipeptin\_biosynthetic\_gene\_cluster | 31 | 77.6407093292 | 384 | 6.1e-106 |
| AAT12283.1 | LtxA | BGC0000384 | Lyngbyatoxin\_biosynthetic\_gene\_cluster | 31 | 80.1079414032 | 383 | 1e-105 |
| BAO84866.1 | putative\_non-ribosomal\_peptide\_synthetase | BGC0000414 | Quinocarcin\_biosynthetic\_gene\_cluster | 31 | 72.8604471858 | 376 | 1.7e-103 |
| CAF32362.1 | putative\_non-ribosomal\_peptide\_synthetase | BGC0000712 | Paromomycin\_biosynthetic\_gene\_cluster | 30 | 80.4934464148 | 367 | 5.9e-101 |
| AAL33758.1 | putative\_non-ribosomal\_peptide\_synthetase | BGC0000421 | Safracin\_biosynthetic\_gene\_cluster | 30 | 72.6291441789 | 353 | 8.8e-97 |
| AAG31130.1 | MxcG | BGC0001345 | Myxochelin\_biosynthetic\_gene\_cluster | 31 | 80.4934464148 | 346 | 1.4e-94 |
| AEA29644.1 | putative\_nonribosomal\_peptide\_synthetase\_and\_kinurenine\_monooxygenase | BGC0000409 | Porothramycin\_biosynthetic\_gene\_cluster | 31 | 75.3276792598 | 342 | 2e-93 |
| ABW71853.1 | nonribosomal\_peptide\_synthetase | BGC0000303 | Anthramycin\_biosynthetic\_gene\_cluster | 30 | 76.5612952968 | 321 | 3.7e-87 |
| ABY83163.1 | Azi25 | BGC0000960 | Azinomycin\_B\_biosynthetic\_gene\_cluster | 30 | 74.5566692367 | 309 | 1.5e-83 |
| AAU34202.1 | mannopeptimycin\_peptide\_synthetase\_MppA | BGC0000388 | Mannopeptimycin\_biosynthetic\_gene\_cluster | 37 | 46.5690053971 | 300 | 6.8e-81 |
| XP\_002384859.1 | NRPS-like\_enzyme,\_putative | BGC0001234\_c2 | 4,4'-piperazine-2,5-diyldimethyl-bis-phenol\_biosynthetic\_gene | 28 | 71.4726291442 | 295 | 2.2e-79 |
| XP\_002384042.1 | NRPS-like\_enzyme,\_putative | BGC0001234 | 4,4'-piperazine-2,5-diyldimethyl-bis-phenol\_biosynthetic\_gene | 27 | 81.2644564379 | 292 | 2.4e-78 |
| AFP87549.1 | NrpS | BGC0001135 | Bicornutin\_biosynthetic\_gene\_cluster | 33 | 48.1881264456 | 276 | 1.4e-73 |
| CAE15637.1 |  | BGC0001128 | Luminmide\_biosynthetic\_gene\_cluster | 34 | 46.3377023901 | 275 | 2.3e-73 |
| ACA09733.2 | fusaricidin\_synthetase | BGC0000358 | Fusaricidin\_biosynthetic\_gene\_cluster | 33 | 46.0292983809 | 272 | 2e-72 |
| CBJ90082.1 | Non\_Ribosomal\_peptide\_synthetase\_(-succinylbenzoate--CoA\_ligase) | BGC0001132 | Xenotetrapeptide\_biosynthetic\_gene\_cluster | 32 | 50.3469545104 | 272 | 2.6e-72 |
| CCJ67637.1 | TaaB | BGC0000447 | Tolaasin\_biosynthetic\_gene\_cluster | 33 | 46.7232074017 | 269 | 1.7e-71 |
| AAU34203.1 | mannopeptimycin\_peptide\_synthetase\_MppB | BGC0000388 | Mannopeptimycin\_biosynthetic\_gene\_cluster | 35 | 48.1110254433 | 268 | 3.7e-71 |
| AEH59100.1 | amino\_acid\_adenylation\_domain-containing\_protein/NRPS | BGC0000385 | Lysobactin\_biosynthetic\_gene\_cluster | 32 | 48.3423284503 | 267 | 6.4e-71 |
| AAY89051.1 | hybrid\_nonribosomal\_peptide\_synthetase/polyketide\_synthase | BGC0001069 | Chivosazole\_biosynthetic\_gene\_cluster | 33 | 50.1927525058 | 267 | 1.1e-70 |
| ABQ96384.2 | fusaricidin\_synthetase | BGC0001152 | Fusaricidin\_biosynthetic\_gene\_cluster | 31 | 48.1110254433 | 266 | 1.4e-70 |
| CDG17981.1 | Non-ribosomal\_peptide\_synthetase | BGC0000464 | Xenoamicins\_biosynthetic\_gene\_cluster | 33 | 49.498843485 | 266 | 1.9e-70 |
| AEI70245.1 | nonribosomal\_peptide\_synthetase\_NRPS | BGC0000401 | Paenibactin\_biosynthetic\_gene\_cluster | 33 | 51.1950655359 | 264 | 9.2e-70 |
| ACS68554.1 | hybrid\_PKS-NRPS\_protein | BGC0001026 | NG-391\_biosynthetic\_gene\_cluster | 29 | 70.8558211257 | 263 | 1.6e-69 |
| AGZ03651.1 | SevB | BGC0000426 | Sevadicin\_biosynthetic\_gene\_cluster | 32 | 50.1156515035 | 260 | 1e-68 |
| AJF34464.1 | Txo2 | BGC0001207 | Teixobactin\_biosynthetic\_gene\_cluster | 33 | 47.8797224364 | 259 | 2.3e-68 |
| ABS74206.1 | FenD | BGC0001095 | Fengycin\_biosynthetic\_gene\_cluster | 32 | 46.800308404 | 259 | 3e-68 |
| EAL85113.2 | hybrid\_PKS-NRPS\_enzyme,\_putative | BGC0001037 | Pseurotin\_A\_biosynthetic\_gene\_cluster | 27 | 76.2528912876 | 258 | 5e-68 |
| AKP45395.1 | CysG | BGC0001413 | Cystobactamide\_biosynthetic\_gene\_cluster | 32 | 46.4919043948 | 258 | 5e-68 |
| CAB38518.1 | CDA\_peptide\_synthetase\_I | BGC0000315 | Calcium-dependent\_antibiotic\_biosynthetic\_gene\_cluster | 32 | 50.6553585197 | 257 | 8.6e-68 |
| CDG17985.1 | Putative\_Ornithine\_racemase\_(fragment) | BGC0000464 | Xenoamicins\_biosynthetic\_gene\_cluster | 33 | 46.1835003855 | 256 | 1.5e-67 |
| AGE25479.1 | putative\_non-ribosomal\_peptide\_synthetase | BGC0001208\_c2 | Poaeamide\_biosynthetic\_gene\_cluster | 32 | 46.800308404 | 256 | 1.9e-67 |
| AGM16412.1 | paenibacterin\_synthetase\_A | BGC0000400 | Paenibacterin\_biosynthetic\_gene\_cluster | 31 | 48.9591364688 | 255 | 2.5e-67 |
| AFJ23825.1 | WLIP\_synthetase\_B | BGC0000462\_c2 | WLIP\_biosynthetic\_gene\_cluster | 31 | 45.7208943716 | 255 | 2.5e-67 |
| CAE52334.1 | non-ribosomal\_peptide\_synthase | BGC0001088 | Albicidin\_biosynthetic\_gene\_cluster | 31 | 50.3469545104 | 255 | 3.3e-67 |
| AEG64698.1 | LpmD | BGC0000379 | Laspartomycin\_biosynthetic\_gene\_cluster | 33 | 48.033924441 | 255 | 4.3e-67 |
| AJF34463.1 | Txo1 | BGC0001207 | Teixobactin\_biosynthetic\_gene\_cluster | 31 | 47.4171164225 | 253 | 9.5e-67 |
| ABH06369.2 | MassC | BGC0000389 | Massetolide\_A\_biosynthetic\_gene\_cluster | 32 | 47.0316114109 | 253 | 1.2e-66 |
| CAJ18237.2 | non-ribosomal\_peptide\_synthetase\_B | BGC0000354 | Friulimicin\_biosynthetic\_gene\_cluster | 34 | 48.3423284503 | 253 | 1.6e-66 |
| AGM16413.1 | paenibacterin\_synthetase\_B | BGC0000400 | Paenibacterin\_biosynthetic\_gene\_cluster | 31 | 46.3377023901 | 252 | 2.1e-66 |
| ALV82356.1 | CDA\_peptide\_synthetase\_I | BGC0001370 | Lipopeptide\_8D1-1\_/\_lipopeptide\_8D1-2\_biosynthetic\_gene\_cluster | 32 | 49.3446414803 | 252 | 2.1e-66 |
| ACZ55942.1 | non-ribosomal\_peptide\_synthetase | BGC0000302 | Anabaenopeptin\_biosynthetic\_gene\_cluster | 32 | 46.800308404 | 252 | 2.1e-66 |
| AAC06346.1 | bacitracin\_synthetase\_1 | BGC0000310 | Bacitracin\_biosynthetic\_gene\_cluster | 31 | 46.0292983809 | 252 | 3.6e-66 |
| AFH75322.1 | nonribosomal\_peptide\_synthetase | BGC0000425 | Sessilin\_biosynthetic\_gene\_cluster | 33 | 48.4194294526 | 251 | 4.7e-66 |
| AAY91421.3 | non-ribosomal\_peptide\_synthetase\_OfaC | BGC0000399 | Orfamide\_biosynthetic\_gene\_cluster | 31 | 49.6530454896 | 250 | 8.1e-66 |
| BAH43766.1 | tyrocidine\_synthetase\_III | BGC0000452 | Tyrocidine\_biosynthetic\_gene\_cluster | 30 | 47.0316114109 | 250 | 8.1e-66 |
| CAQ71828.1 | non\_ribosomal\_peptide\_synthase,\_antibiotic\_synthesis;\_contains\_3\_condensation\_domains,\_2\_AMP-acid\_ligases\_II\_domains,\_2\_PP-binding,\_Phosphopantetheine\_attachment\_site | BGC0001189 | Taiwachelin\_biosynthetic\_gene\_cluster | 34 | 47.4171164225 | 250 | 1.4e-65 |
| ABI26078.1 | OciB | BGC0000331 | Cyanopeptin\_biosynthetic\_gene\_cluster | 32 | 47.1858134156 | 249 | 1.8e-65 |
| AEW31019.1 | plipastatin\_synthetase | BGC0000407 | Plipastatin\_biosynthetic\_gene\_cluster | 31 | 47.4942174248 | 248 | 5.2e-65 |
| AIW82284.1 | PuwG | BGC0001125 | Puwainaphycins\_biosynthetic\_gene\_cluster | 30 | 49.6530454896 | 247 | 8.9e-65 |
| AHB82071.1 | non\_ribosomal\_peptide\_synthetase | BGC0001231 | Microsclerodermins\_biosynthetic\_gene\_cluster | 32 | 51.5034695451 | 246 | 1.5e-64 |
| ABW17377.1 | PsoC | BGC0000411 | Putisolvin\_biosynthetic\_gene\_cluster | 31 | 51.2721665382 | 246 | 2e-64 |
| AHH53506.1 | non-ribosomal\_peptide\_synthetase | BGC0000439 | Taromycin\_biosynthetic\_gene\_cluster | 33 | 48.1110254433 | 245 | 3.4e-64 |
| CDG17980.1 | Putative\_Ornithine\_racemase\_(fragment) | BGC0000464 | Xenoamicins\_biosynthetic\_gene\_cluster | 32 | 47.1087124133 | 245 | 3.4e-64 |
| BAH22764.1 | nonribosomal\_peptide\_synthetase | BGC0001018 | Micropeptin\_biosynthetic\_gene\_cluster | 31 | 47.0316114109 | 245 | 4.4e-64 |
| BAC67536.1 | arthrofactin\_synthetase\_C | BGC0000305 | Arthrofactin\_biosynthetic\_gene\_cluster | 30 | 52.1973785659 | 245 | 4.4e-64 |
| AAZ03552.1 | McnC | BGC0000332 | Cyanopeptolin\_biosynthetic\_gene\_cluster | 32 | 47.1087124133 | 245 | 4.4e-64 |
| AEW31021.1 | plipastatin\_synthetase | BGC0000407 | Plipastatin\_biosynthetic\_gene\_cluster | 31 | 47.8797224364 | 243 | 1.3e-63 |
| CAD17792.1 | probable\_non\_ribosomal\_peptide\_synthetase\_protein | BGC0001363 | Ralsolamycin\_biosynthetic\_gene\_cluster | 33 | 48.6507324595 | 242 | 2.2e-63 |
| AAZ55899.1 | amino\_acid\_adenylation | BGC0000359 | Fuscachelin\_biosynthetic\_gene\_cluster | 33 | 48.6507324595 | 242 | 2.9e-63 |
| ABS74207.1 | FenC | BGC0001095 | Fengycin\_biosynthetic\_gene\_cluster | 30 | 48.1881264456 | 242 | 2.9e-63 |
| ABS74209.1 | FenA | BGC0001095 | Fengycin\_biosynthetic\_gene\_cluster | 30 | 48.265227448 | 242 | 2.9e-63 |
| ADG27358.1 | peptide\_synthetase | BGC0000296 | Actinomycin\_biosynthetic\_gene\_cluster | 31 | 49.1133384734 | 242 | 2.9e-63 |
| BAP05597.1 | CalI | BGC0000967 | Calyculin\_biosynthetic\_gene\_cluster | 31 | 47.1858134156 | 242 | 3.7e-63 |
| AFJ23826.1 | WLIP\_synthetase\_C | BGC0000462\_c2 | WLIP\_biosynthetic\_gene\_cluster | 32 | 45.8750963763 | 242 | 3.7e-63 |
| ABL74936.1 | TlmX | BGC0001048 | Tallysomycin\_biosynthetic\_gene\_cluster | 32 | 48.265227448 | 241 | 6.4e-63 |
| AFJ14794.1 | PlpE | BGC0000403 | Pelgipeptin\_biosynthetic\_gene\_cluster | 30 | 46.3377023901 | 241 | 6.4e-63 |
| KPN90376.1 | NupC | BGC0001416 | Nunapeptin\_/\_nunamycin\_biosynthetic\_gene\_cluster | 32 | 50.8095605243 | 240 | 8.3e-63 |
| AJM89735.1 | PmxA | BGC0001192 | Colistin\_biosynthetic\_gene\_cluster | 31 | 45.9521973786 | 240 | 8.3e-63 |
| AHF21228.1 | TriD | BGC0000449 | Tridecaptin\_biosynthetic\_gene\_cluster | 30 | 46.4148033924 | 240 | 1.4e-62 |
| ABF87031.1 | non-ribosomal\_peptide\_synthetase/polyketide\_synthase | BGC0000393 | Myxoprincomide\_biosynthetic\_gene\_cluster | 31 | 48.8049344641 | 239 | 1.9e-62 |
| AHB82059.1 | non\_ribosomal\_peptide\_synthetase/polyketide\_synthase | BGC0001019 | Microsclerodermins\_biosynthetic\_gene\_cluster | 31 | 47.2629144179 | 239 | 1.9e-62 |
| AAG02355.1 | peptide\_synthetase\_NRPS9-8 | BGC0000963 | Bleomycin\_biosynthetic\_gene\_cluster | 33 | 46.7232074017 | 239 | 2.4e-62 |
| AEH59099.1 | amino\_acid\_adenylation\_domain-containing\_protein/NRPS | BGC0000385 | Lysobactin\_biosynthetic\_gene\_cluster | 32 | 48.3423284503 | 238 | 3.2e-62 |
| BAH22765.1 | nonribosomal\_peptide\_synthetase | BGC0001018 | Micropeptin\_biosynthetic\_gene\_cluster | 30 | 47.9568234387 | 238 | 3.2e-62 |
| KPN93063.1 | NupA | BGC0001416 | Nunapeptin\_/\_nunamycin\_biosynthetic\_gene\_cluster | 34 | 46.4148033924 | 238 | 4.1e-62 |
| ABW17375.1 | PsoA | BGC0000411 | Putisolvin\_biosynthetic\_gene\_cluster | 31 | 47.0316114109 | 238 | 4.1e-62 |
| EFE73313.1 | nonribosomal\_peptide\_synthetase | BGC0000431 | Stenothricin\_biosynthetic\_gene\_cluster | 31 | 47.2629144179 | 238 | 5.4e-62 |
| ALD82526.1 | non-ribosomal\_peptide\_synthase | BGC0001212 | Nannocystin\_biosynthetic\_gene\_cluster | 34 | 46.0292983809 | 238 | 5.4e-62 |
| ABD65956.1 | nonribosomal\_peptide\_synthetase | BGC0000341 | Enduracidin\_biosynthetic\_gene\_cluster | 32 | 47.3400154202 | 238 | 5.4e-62 |
| AEU11006.1 | NpnB | BGC0001029 | Nostophycin\_biosynthetic\_gene\_cluster | 31 | 45.9521973786 | 238 | 5.4e-62 |
| CDF96614.1 | Long-chain-fatty-acid--CoA\_ligase | BGC0001149 | Brabantamide\_biosynthetic\_gene\_cluster | 30 | 49.267540478 | 238 | 5.4e-62 |
| AEW31022.1 | plipastatin\_synthetase | BGC0000407 | Plipastatin\_biosynthetic\_gene\_cluster | 31 | 47.6484194295 | 237 | 7.1e-62 |
| CAM56770.1 |  | BGC0000354 | Friulimicin\_biosynthetic\_gene\_cluster | 32 | 46.800308404 | 237 | 7.1e-62 |
| AAT09805.1 | NocB | BGC0000395 | Nocardicin\_A\_biosynthetic\_gene\_cluster | 34 | 47.1087124133 | 237 | 7.1e-62 |
| AGM16414.1 | paenibacterin\_synthetase\_C | BGC0000400 | Paenibacterin\_biosynthetic\_gene\_cluster | 30 | 45.8750963763 | 237 | 1.2e-61 |
| CAQ71829.1 | non\_ribosomal\_peptide\_synthase,\_antibiotic\_synthesis;\_contains\_4\_condensation\_domains,\_3\_AMP-acid\_ligases\_II\_domains,\_3\_PP-binding,\_Phosphopantetheine\_attachment\_site\_and\_a\_putative\_thioesterase\_domain | BGC0001189 | Taiwachelin\_biosynthetic\_gene\_cluster | 33 | 48.7278334618 | 237 | 1.2e-61 |
| AIW82282.1 | PuwE | BGC0001125 | Puwainaphycins\_biosynthetic\_gene\_cluster | 31 | 45.7979953739 | 237 | 1.2e-61 |
| ANQ43347.1 | non-ribosomal\_peptide\_synthetase | BGC0001347 | Poaeamide\_B\_biosynthetic\_gene\_cluster | 33 | 48.3423284503 | 236 | 2.1e-61 |
| ACY06285.1 | non-ribosomal\_peptide\_synthetase | BGC0001042 | Sanglifehrin\_A\_biosynthetic\_gene\_cluster | 32 | 47.1858134156 | 236 | 2.1e-61 |
| AAX31559.1 | peptide\_synthetase\_3 | BGC0000336 | Daptomycin\_biosynthetic\_gene\_cluster | 34 | 48.4965304549 | 235 | 2.7e-61 |
| AHB82072.1 | non\_ribosomal\_peptide\_synthetase/polyketide\_synthase | BGC0001231 | Microsclerodermins\_biosynthetic\_gene\_cluster | 30 | 50.1927525058 | 235 | 3.5e-61 |
| BAH43871.1 | truncated\_linear\_pentadecapeptide\_gramicidin\_synthetase\_LgrC | BGC0000367 | Gramicidin\_biosynthetic\_gene\_cluster | 30 | 48.4965304549 | 235 | 3.5e-61 |
| BAF50711.1 | non\_ribosomal\_peptide\_synthetase\_for\_virginiamycin\_S | BGC0001116 | Virginiamycin\_biosynthetic\_gene\_cluster | 32 | 47.8797224364 | 234 | 6e-61 |
| AFH75329.1 | nonribosomal\_peptide\_synthetase | BGC0000398 | Orfamide\_biosynthetic\_gene\_cluster | 31 | 48.8820354665 | 234 | 7.8e-61 |
| AIW82283.1 | PuwF | BGC0001125 | Puwainaphycins\_biosynthetic\_gene\_cluster | 29 | 49.6530454896 | 234 | 7.8e-61 |
| CAM59606.1 | non-ribosomal\_peptide\_synthetase | BGC0000297 | Aeruginoside\_biosynthetic\_gene\_cluster | 30 | 48.6507324595 | 233 | 1e-60 |
| AAY37655.1 | Amino\_acid\_adenylation | BGC0000437 | Syringomycin\_biosynthetic\_gene\_cluster | 29 | 50.1156515035 | 233 | 1e-60 |
| AAO72425.1 | syringopeptin\_synthetase\_C | BGC0000438 | Syringopeptin\_biosynthetic\_gene\_cluster | 29 | 50.1156515035 | 233 | 1.7e-60 |
| AAF99707.2 | syringopeptin\_synthetase | BGC0000438 | Syringopeptin\_biosynthetic\_gene\_cluster | 29 | 59.2135697764 | 232 | 2.3e-60 |
| AAY37653.1 | Amino\_acid\_adenylation | BGC0000437 | Syringomycin\_biosynthetic\_gene\_cluster | 29 | 59.2135697764 | 232 | 2.3e-60 |
| ACM68684.1 | AerB | BGC0000298 | Aeruginosin\_biosynthetic\_gene\_cluster | 29 | 49.267540478 | 232 | 2.3e-60 |
| AEZ51516.1 | PmxA | BGC0001153 | Polymyxin\_biosynthetic\_gene\_cluster | 31 | 45.7208943716 | 232 | 3e-60 |
| CAE14171.1 |  | BGC0000383 | Luminmycin\_biosynthetic\_gene\_cluster | 29 | 48.4965304549 | 232 | 3e-60 |
| AIS24862.1 | Dst18 | BGC0001147 | Distamycin\_biosynthetic\_gene\_cluster | 32 | 50.4240555127 | 232 | 3e-60 |
| ACU36654.1 | amino\_acid\_adenylation\_domain\_protein | BGC0000392 | Mirubactin\_biosynthetic\_gene\_cluster | 32 | 47.2629144179 | 232 | 3e-60 |
| ABX37382.1 | amino\_acid\_adenylation\_domain\_protein | BGC0000984 | Delftibactin\_biosynthetic\_gene\_cluster | 31 | 48.1110254433 | 232 | 3e-60 |
| AHZ34238.1 | CipA | BGC0001389 | Cichopeptin\_biosynthetic\_gene\_cluster | 31 | 49.1133384734 | 232 | 3.9e-60 |
| sln14 | nonribosomal\_peptide\_synthetase/polyketide\_synthase\_type\_I | BGC0001230 | Salinamides\_biosynthetic\_gene\_cluster | 31 | 48.8820354665 | 232 | 3.9e-60 |
| AFH75320.1 | nonribosomal\_peptide\_synthetase | BGC0000425 | Sessilin\_biosynthetic\_gene\_cluster | 31 | 49.8072474942 | 232 | 3.9e-60 |
| AEG64697.1 | LpmC | BGC0000379 | Laspartomycin\_biosynthetic\_gene\_cluster | 31 | 47.0316114109 | 231 | 5.1e-60 |
| AHB82058.1 | non\_ribosomal\_peptide\_synthetase | BGC0001019 | Microsclerodermins\_biosynthetic\_gene\_cluster | 32 | 51.966075559 | 231 | 6.6e-60 |
| AAT09804.1 | NocA | BGC0000395 | Nocardicin\_A\_biosynthetic\_gene\_cluster | 32 | 45.4895913647 | 230 | 1.5e-59 |
| AAZ03554.1 | McnE | BGC0000332 | Cyanopeptolin\_biosynthetic\_gene\_cluster | 30 | 47.2629144179 | 230 | 1.5e-59 |
| ABD65958.1 | nonribosomal\_peptide\_synthetase | BGC0000341 | Enduracidin\_biosynthetic\_gene\_cluster | 32 | 46.4919043948 | 229 | 1.9e-59 |
| AHZ20784.1 | non-ribosomal\_peptide\_synthase | BGC0000369 | Hassallidins\_biosynthetic\_gene\_cluster | 30 | 46.2606013878 | 229 | 1.9e-59 |
| AAO23333.1 | NcpA | BGC0000397 | Nostocyclopeptide\_biosynthetic\_gene\_cluster | 31 | 47.0316114109 | 229 | 2.5e-59 |
| AEU11003.1 | NpnC | BGC0001029 | Nostophycin\_biosynthetic\_gene\_cluster | 28 | 55.7440246723 | 228 | 3.3e-59 |
| AAZ03550.1 | McnA | BGC0000332 | Cyanopeptolin\_biosynthetic\_gene\_cluster | 30 | 44.9498843485 | 228 | 3.3e-59 |
| AHZ34243.1 | CipF | BGC0001389 | Cichopeptin\_biosynthetic\_gene\_cluster | 31 | 49.0362374711 | 228 | 3.3e-59 |
| AFJ23819.1 | WLIP\_synthetase\_A | BGC0000462 | WLIP\_biosynthetic\_gene\_cluster | 31 | 46.6461063994 | 228 | 4.3e-59 |
| AAF17280.1 | NosC | BGC0001028 | Nostopeptolide\_biosynthetic\_gene\_cluster | 31 | 47.1858134156 | 227 | 7.3e-59 |
| AHZ34239.1 | CipB | BGC0001389 | Cichopeptin\_biosynthetic\_gene\_cluster | 31 | 49.0362374711 | 227 | 7.3e-59 |
| ACA97576.1 | PmxA | BGC0000408 | Polymyxin\_biosynthetic\_gene\_cluster | 30 | 46.0292983809 | 227 | 7.3e-59 |
| AHF21229.1 | TriE | BGC0000449 | Tridecaptin\_biosynthetic\_gene\_cluster | 30 | 47.4942174248 | 227 | 7.3e-59 |
| CAJ46692.1 | non\_ribosomal\_peptide\_synthase | BGC0000969 | Chondramide\_biosynthetic\_gene\_cluster | 32 | 48.6507324595 | 227 | 7.3e-59 |
| CDG17982.1 | Non-ribosomal\_peptide\_synthetase | BGC0000464 | Xenoamicins\_biosynthetic\_gene\_cluster | 30 | 49.0362374711 | 227 | 9.5e-59 |
| KPN93064.1 | NupB | BGC0001416 | Nunapeptin\_/\_nunamycin\_biosynthetic\_gene\_cluster | 33 | 46.5690053971 | 227 | 1.2e-58 |
| CCJ67640.1 | TaaE | BGC0000447 | Tolaasin\_biosynthetic\_gene\_cluster | 30 | 47.3400154202 | 226 | 1.6e-58 |
| ABS74180.1 | BmyB | BGC0001090 | Bacillomycin\_biosynthetic\_gene\_cluster | 30 | 46.3377023901 | 226 | 2.1e-58 |
| ALV86866.1 | Tlo20 | BGC0001406 | Telomycin\_biosynthetic\_gene\_cluster | 31 | 46.3377023901 | 226 | 2.1e-58 |
| ALV86867.1 | Tlo21 | BGC0001406 | Telomycin\_biosynthetic\_gene\_cluster | 31 | 46.7232074017 | 225 | 2.8e-58 |
| AAY37647.1 | Amino\_acid\_adenylation | BGC0000437 | Syringomycin\_biosynthetic\_gene\_cluster | 31 | 45.7208943716 | 225 | 2.8e-58 |
| ABD65957.1 | nonribosomal\_peptide\_synthetase | BGC0000341 | Enduracidin\_biosynthetic\_gene\_cluster | 31 | 48.8049344641 | 225 | 2.8e-58 |
| AGS77307.1 | NRPS\_modules\_1-2 | BGC0001178 | UK-68,597\_biosynthetic\_gene\_cluster | 30 | 45.9521973786 | 225 | 2.8e-58 |
| AHZ20774.1 | non-ribosomal\_peptide\_synthase | BGC0000369 | Hassallidins\_biosynthetic\_gene\_cluster | 30 | 48.3423284503 | 225 | 2.8e-58 |
| EWS95124.1 | hypothetical\_protein | BGC0000306 | Arylomycin\_biosynthetic\_gene\_cluster | 30 | 50.3469545104 | 225 | 2.8e-58 |
| AAO56329.1 | non-ribosomal\_peptide\_synthetase\_SyfB | BGC0000435 | Syringafactin\_biosynthetic\_gene\_cluster | 29 | 48.6507324595 | 225 | 4.7e-58 |
| AAS98786.1 | JamO | BGC0001001 | Jamaicamide\_biosynthetic\_gene\_cluster | 30 | 47.2629144179 | 225 | 4.7e-58 |
| AHZ34233.1 | CifB | BGC0000323 | Cichofactin\_biosynthetic\_gene\_cluster | 30 | 49.1904394757 | 225 | 4.7e-58 |
| AAM80539.1 | StaA | BGC0000290 | A47934\_biosynthetic\_gene\_cluster | 30 | 44.9498843485 | 224 | 6.2e-58 |
| AAY91420.2 | non-ribosomal\_peptide\_synthetase\_OfaB | BGC0000399 | Orfamide\_biosynthetic\_gene\_cluster | 31 | 48.4965304549 | 224 | 6.2e-58 |
| AAF00961.1 | McyB | BGC0001017 | Microcystin\_biosynthetic\_gene\_cluster | 31 | 46.2606013878 | 224 | 8.1e-58 |
| CBZ42146.1 | putative\_non-ribosomal\_peptide\_synthetase | BGC0001117 | Himastatin\_biosynthetic\_gene\_cluster | 31 | 48.6507324595 | 224 | 8.1e-58 |
| AAF15891.2 | NosA | BGC0001028 | Nostopeptolide\_biosynthetic\_gene\_cluster | 30 | 47.3400154202 | 223 | 1.4e-57 |
| CBW54672.1 | non\_ribosomal\_peptide\_synthetase | BGC0000971 | Cinnabaramide\_biosynthetic\_gene\_cluster | 31 | 45.9521973786 | 223 | 1.4e-57 |
| AAY32966.1 | DszC | BGC0001093 | Disorazole\_biosynthetic\_gene\_cluster | 31 | 49.7301464919 | 223 | 1.8e-57 |
| AHZ34242.1 | CipE | BGC0001389 | Cichopeptin\_biosynthetic\_gene\_cluster | 31 | 46.4919043948 | 223 | 1.8e-57 |
| ABW17376.1 | PsoB | BGC0000411 | Putisolvin\_biosynthetic\_gene\_cluster | 30 | 46.0292983809 | 222 | 2.3e-57 |
| AFH75321.1 | nonribosomal\_peptide\_synthetase | BGC0000425 | Sessilin\_biosynthetic\_gene\_cluster | 30 | 47.3400154202 | 222 | 2.3e-57 |
| ABH06368.2 | MassB | BGC0000389 | Massetolide\_A\_biosynthetic\_gene\_cluster | 30 | 49.8843484965 | 222 | 2.3e-57 |
| AHI59109.1 | locillomycin\_synthase\_B | BGC0001005 | Locillomycin\_biosynthetic\_gene\_cluster | 27 | 47.2629144179 | 222 | 2.3e-57 |
| ACG60782.1 | NRPS(C/A/PCP/C/A/PCP) | BGC0001058 | Zorbamycin\_biosynthetic\_gene\_cluster | 31 | 47.4171164225 | 222 | 2.3e-57 |
| CAE02631.1 | surfactin\_synthetase\_B | BGC0000433 | Surfactin\_biosynthetic\_gene\_cluster | 29 | 45.566692367 | 222 | 3.1e-57 |
| ACZ55945.1 | non-ribosomal\_peptide\_synthetase | BGC0000302 | Anabaenopeptin\_biosynthetic\_gene\_cluster | 30 | 47.7255204318 | 222 | 4e-57 |
| ANQ43349.1 | non-ribosomal\_peptide\_synthetase | BGC0001347\_c2 | Poaeamide\_B\_biosynthetic\_gene\_cluster | 30 | 46.9545104086 | 222 | 4e-57 |
| CBJ79916.1 | putative\_Ornithine\_racemase | BGC0001133 | Taxlllaid\_biosynthetic\_gene\_cluster | 29 | 46.9545104086 | 221 | 5.2e-57 |
| AGM14933.1 | xantholysin\_synthetase\_B | BGC0000463 | Xantholysin\_biosynthetic\_gene\_cluster | 31 | 48.3423284503 | 221 | 6.8e-57 |
| AHH53507.1 | non-ribosomal\_peptide\_synthetase | BGC0000439 | Taromycin\_biosynthetic\_gene\_cluster | 32 | 51.1950655359 | 221 | 6.8e-57 |
| ABV56588.1 | KtzH | BGC0000378 | Kutznerides\_biosynthetic\_gene\_cluster | 31 | 53.3538936006 | 221 | 6.8e-57 |
| BAH43765.1 | tyrocidine\_synthetase\_II | BGC0000452 | Tyrocidine\_biosynthetic\_gene\_cluster | 29 | 47.3400154202 | 221 | 6.8e-57 |
| AAU39360.1 | lichenysin\_synthase\_LchAB | BGC0000381 | Lichenysin\_biosynthetic\_gene\_cluster | 31 | 46.1835003855 | 220 | 8.9e-57 |
| CAQ34921.1 | nonribosomal\_peptide\_synthetase | BGC0000986 | Dkxanthene\_biosynthetic\_gene\_cluster | 31 | 48.8820354665 | 220 | 8.9e-57 |
| CAD91220.1 | putative\_non-ribosomal\_peptide\_synthetase,\_modules\_1-2 | BGC0000289 | A40926\_biosynthetic\_gene\_cluster | 30 | 49.4217424827 | 220 | 1.2e-56 |
| AAO56328.1 | non-ribosomal\_peptide\_synthetase\_SyfA | BGC0000435 | Syringafactin\_biosynthetic\_gene\_cluster | 29 | 46.8774094063 | 220 | 1.5e-56 |
| AFJ14795.1 | PlpF | BGC0000403 | Pelgipeptin\_biosynthetic\_gene\_cluster | 29 | 47.8026214341 | 220 | 1.5e-56 |
| ABL74939.1 | TlmVII | BGC0001048 | Tallysomycin\_biosynthetic\_gene\_cluster | 31 | 51.734772552 | 219 | 2e-56 |
| BAB69699.1 | iturin\_A\_synthetase\_B | BGC0001098 | Iturin\_biosynthetic\_gene\_cluster | 29 | 46.3377023901 | 219 | 2.6e-56 |
| AAK81827.1 | peptide\_synthetase | BGC0000326 | Complestatin\_biosynthetic\_gene\_cluster | 31 | 46.8774094063 | 218 | 3.4e-56 |
| AHZ20781.1 | non-ribosomal\_peptide\_synthase | BGC0000369 | Hassallidins\_biosynthetic\_gene\_cluster | 30 | 49.0362374711 | 218 | 3.4e-56 |
| AHB38497.1 | EpxD | BGC0000346 | Epoxomicin\_biosynthetic\_gene\_cluster | 35 | 37.5481881264 | 218 | 4.4e-56 |
| AHI59108.1 | locillomycin\_synthase\_A | BGC0001005 | Locillomycin\_biosynthetic\_gene\_cluster | 29 | 46.3377023901 | 218 | 4.4e-56 |
| CAE53350.1 | non-ribosomal\_peptide\_synthetase | BGC0000440 | Teicoplanin\_biosynthetic\_gene\_cluster | 32 | 44.4101773323 | 218 | 5.8e-56 |
| CAG15009.1 | peptide\_synthetase,\_module\_1-2 | BGC0000441 | Teicoplanin\_biosynthetic\_gene\_cluster | 32 | 44.4101773323 | 218 | 5.8e-56 |
| ABO15860.1 | polyketide\_synthase | BGC0000130 | Pyrrolomycin\_biosynthetic\_gene\_cluster | 32 | 37.3939861218 | 217 | 7.6e-56 |
| ABO15888.1 | polyketide\_synthase | BGC0000132 | Pyrrolomycin\_biosynthetic\_gene\_cluster | 34 | 36.1603700848 | 217 | 7.6e-56 |
| BAH43764.1 | tyrocidine\_synthetase\_I | BGC0000452 | Tyrocidine\_biosynthetic\_gene\_cluster | 29 | 46.1835003855 | 217 | 9.9e-56 |
| CAE02633.1 | surfactin\_synthetase\_C | BGC0000433 | Surfactin\_biosynthetic\_gene\_cluster | 29 | 46.5690053971 | 216 | 1.7e-55 |
| AAF17281.1 | NosD | BGC0001028 | Nostopeptolide\_biosynthetic\_gene\_cluster | 30 | 47.1858134156 | 216 | 1.7e-55 |
| CAJ88610.1 | putative\_non-ribosomal\_peptide\_synthetase | BGC0000327 | Congocidine\_biosynthetic\_gene\_cluster | 34 | 47.6484194295 | 216 | 1.7e-55 |
| AGE26255.1 | putative\_non-ribosomal\_peptide\_synthetase | BGC0001208 | Poaeamide\_biosynthetic\_gene\_cluster | 31 | 48.265227448 | 216 | 2.2e-55 |
| ACG60776.1 | NRPS(AL/ACP/C/A/PCP/C/A) | BGC0001058 | Zorbamycin\_biosynthetic\_gene\_cluster | 31 | 46.1835003855 | 216 | 2.2e-55 |
| CAY48789.1 | putative\_non-ribosomal\_peptide\_synthetase | BGC0001312 | Viscosin\_biosynthetic\_gene\_cluster | 31 | 47.2629144179 | 215 | 3.7e-55 |
| CAJ96470.1 | Non-ribosomal\_peptide\_synthetase | BGC0000330 | Cupriachelin\_biosynthetic\_gene\_cluster | 30 | 46.7232074017 | 215 | 4.9e-55 |
| KFH48607.1 | N-like\_protein | BGC0000317 | Cephalosporin\_biosynthetic\_gene\_cluster | 29 | 47.1087124133 | 215 | 4.9e-55 |
| ABA70582.1 | alpha-aminoadypil-cysteinyl-valine\_synthetase | BGC0000404 | Penicillin\_biosynthetic\_gene\_cluster | 31 | 48.1110254433 | 215 | 4.9e-55 |
| ABR12615.1 | ACV\_synthetase | BGC0000405 | Penicillin\_biosynthetic\_gene\_cluster | 31 | 48.1110254433 | 215 | 4.9e-55 |
| ABV99085.1 | thioester\_reductase\_domain | BGC0001007 | Lymphostin\_biosynthetic\_gene\_cluster | 33 | 38.6276021588 | 215 | 4.9e-55 |
| AAC06347.1 | bacitracin\_synthetase\_2 | BGC0000310 | Bacitracin\_biosynthetic\_gene\_cluster | 28 | 48.5736314572 | 213 | 1.1e-54 |
| AAK81826.1 | peptide\_synthetase | BGC0000326 | Complestatin\_biosynthetic\_gene\_cluster | 36 | 35.2351580571 | 213 | 1.1e-54 |
| AKA59447.1 | non-ribosomal\_peptide\_synthetase | BGC0001203 | Clarexpoxcin\_biosynthetic\_gene\_cluster | 30 | 47.9568234387 | 213 | 1.1e-54 |
| AAY93356.2 | non-ribosomal\_peptide\_synthetase\_PvdI | BGC0000413 | Pyoverdine\_biosynthetic\_gene\_cluster | 30 | 47.8026214341 | 213 | 1.4e-54 |
| BAH43870.1 | putative\_linear\_pentadecapeptide\_gramicidin\_synthetase\_LgrB | BGC0000367 | Gramicidin\_biosynthetic\_gene\_cluster | 29 | 48.3423284503 | 213 | 1.9e-54 |
| ABP55493.1 | thioester\_reductase\_domain | BGC0001006 | Lymphostin\_biosynthetic\_gene\_cluster | 33 | 37.7023901311 | 212 | 2.4e-54 |
| ABG00046.1 | non-ribosomal\_peptide\_synthetase | BGC0000387 | Mangotoxin\_biosynthetic\_gene\_cluster | 25 | 69.8535080956 | 211 | 5.4e-54 |
| AHI59110.1 | locillomycin\_synthase\_C | BGC0001005 | Locillomycin\_biosynthetic\_gene\_cluster | 28 | 45.566692367 | 211 | 5.4e-54 |
| AHZ34232.1 | CifA | BGC0000323 | Cichofactin\_biosynthetic\_gene\_cluster | 30 | 46.800308404 | 210 | 9.2e-54 |
| AID65224.1 | nonribosomal\_peptide\_synthetase | BGC0000335 | Cystomanamides\_biosynthetic\_gene\_cluster | 29 | 47.3400154202 | 210 | 1.6e-53 |
| AOA33122.1 | nonribosomal\_peptide\_synthetase | BGC0001346 | Bananamides\_biosynthetic\_gene\_cluster | 31 | 46.1835003855 | 210 | 1.6e-53 |
| ABV79985.1 | ApnA | BGC0000301 | Anabaenopeptin\_biosynthetic\_gene\_cluster | 29 | 46.3377023901 | 209 | 2.7e-53 |
| BAO84868.1 | putative\_non-ribosomal\_peptide\_synthetase | BGC0000414 | Quinocarcin\_biosynthetic\_gene\_cluster | 32 | 40.0154202005 | 208 | 3.5e-53 |
| AAO62586.1 | peptide\_sythetase | BGC0001016 | Microcystin\_biosynthetic\_gene\_cluster | 30 | 44.4101773323 | 208 | 4.6e-53 |
| ABD14711.1 | CesA | BGC0000320 | Cereulide\_biosynthetic\_gene\_cluster | 29 | 44.0246723207 | 208 | 6e-53 |
| CAD29797.1 | peptide\_synthetase | BGC0001015 | Microcystin\_biosynthetic\_gene\_cluster | 30 | 46.1835003855 | 207 | 1e-52 |
| CAE02630.1 | surfactin\_synthetase\_A | BGC0000433 | Surfactin\_biosynthetic\_gene\_cluster | 28 | 49.9614494988 | 207 | 1.3e-52 |
| BAT51067.1 | type\_I\_polyketide\_synthase | BGC0001296 | Streptazone\_E\_biosynthetic\_gene\_cluster | 30 | 42.0971472629 | 206 | 1.7e-52 |
| ALP32042.1 | CycB | BGC0001293 | Cyclizidine\_biosynthetic\_gene\_cluster | 32 | 37.933693138 | 206 | 2.3e-52 |
| CAC11137.1 | NikP1\_protein | BGC0000876 | Nikkomycin\_biosynthetic\_gene\_cluster | 31 | 50.4240555127 | 205 | 3e-52 |
| CAJ34367.1 | NRPS\_protein | BGC0000445 | Thiocoraline\_biosynthetic\_gene\_cluster | 30 | 48.1110254433 | 205 | 5.1e-52 |
| AAY93445.1 | non-ribosomal\_peptide\_synthetase\_PvdL | BGC0000413 | Pyoverdine\_biosynthetic\_gene\_cluster | 29 | 44.8727833462 | 204 | 6.6e-52 |
| ANQ43348.1 | non-ribosomal\_peptide\_synthetase | BGC0001347\_c2 | Poaeamide\_B\_biosynthetic\_gene\_cluster | 30 | 50.732459522 | 204 | 8.6e-52 |
| CAA11795.1 | PCZA363.4 | BGC0000322 | Chloroeremomycin\_biosynthetic\_gene\_cluster | 30 | 44.2559753277 | 204 | 8.6e-52 |
| CAC22144.1 | putative\_type\_I\_polyketide\_synthase | BGC0000038 | Coelimycin\_biosynthetic\_gene\_cluster | 31 | 36.1603700848 | 204 | 8.6e-52 |
| BAO84861.1 | putative\_non-ribosomal\_peptide\_synthetase | BGC0000414 | Quinocarcin\_biosynthetic\_gene\_cluster | 29 | 45.8750963763 | 203 | 1.1e-51 |
| ADY76667.1 | non-ribosomal\_peptide\_synthetase | BGC0000950 | Napsamycin\_biosynthetic\_gene\_cluster | 29 | 48.1110254433 | 203 | 1.5e-51 |
| AGA37267.1 | NRPS | BGC0000816 | Malbrancheamide\_biosynthetic\_gene\_cluster | 26 | 71.0100231303 | 203 | 1.5e-51 |
| AGJ76605.1 | HglB | BGC0000869 | Heterocyst\_glycolipids\_biosynthetic\_gene\_cluster | 36 | 29.298380879 | 202 | 2.5e-51 |
| AFP87523.1 | type\_I\_polyketide\_synthase | BGC0001159 | Marinopyrrole\_biosynthetic\_gene\_cluster | 31 | 37.933693138 | 201 | 4.3e-51 |
| ANI24100.1 | nonribosomal\_peptide\_synthetase | BGC0001235 | Antalid\_biosynthetic\_gene\_cluster | 30 | 47.8797224364 | 201 | 5.6e-51 |
| AAZ23078.1 | peptide\_synthetase | BGC0000291 | A54145\_biosynthetic\_gene\_cluster | 32 | 48.7278334618 | 201 | 5.6e-51 |
| AEP18655.1 | WAPS2 | BGC0000461 | WAP-8294A2\_(lotilibcin)\_biosynthetic\_gene\_cluster | 33 | 35.2351580571 | 201 | 7.3e-51 |
| CAJ77696.1 | MPS2\_protein | BGC0000363 | Glycopeptidolipid\_biosynthetic\_gene\_cluster | 29 | 49.3446414803 | 200 | 9.6e-51 |
| CAY48788.1 | putative\_non-ribosomal\_peptide\_synthetase | BGC0001312 | Viscosin\_biosynthetic\_gene\_cluster | 30 | 46.8774094063 | 200 | 1.2e-50 |
| AGC09528.1 | NRPS | BGC0001183 | Lobophorin\_biosynthetic\_gene\_cluster | 31 | 40.5551272167 | 200 | 1.6e-50 |
| AFK57215.1 | DidD | BGC0000985 | Didemnin\_biosynthetic\_gene\_cluster | 30 | 47.4171164225 | 200 | 1.6e-50 |
| AAG02356.1 | peptide\_synthetase\_NRPS7 | BGC0000963 | Bleomycin\_biosynthetic\_gene\_cluster | 30 | 47.6484194295 | 199 | 2.1e-50 |
| CAJ77716.1 | Mps2\_protein | BGC0000364 | Glycopeptidolipid\_biosynthetic\_gene\_cluster | 28 | 48.4965304549 | 198 | 6.2e-50 |
| ADN26252.1 | peptide\_synthetase | BGC0000951 | Pacidamycin\_biosynthetic\_gene\_cluster | 28 | 46.9545104086 | 197 | 8.1e-50 |
| CBJ79915.1 | putative\_Phenylalanine\_racemase\_(ATP-hydrolyzing) | BGC0001133 | Taxlllaid\_biosynthetic\_gene\_cluster | 28 | 46.2606013878 | 197 | 8.1e-50 |
| AGD80623.1 | non-ribosomal\_peptide\_synthetase | BGC0000394 | Naphthyridinomycin\_biosynthetic\_gene\_cluster | 31 | 44.4101773323 | 196 | 1.4e-49 |
| ABP57748.1 | DepD | BGC0000993 | FK228\_biosynthetic\_gene\_cluster | 30 | 47.3400154202 | 196 | 1.4e-49 |
| EDY47118.1 | N-(5-amino-5-carboxypentanoyl)-L-cysteinyl-D-\_valine\_synthase | BGC0000319 | Cephamycin\_C\_biosynthetic\_gene\_cluster | 28 | 50.3469545104 | 195 | 3.1e-49 |
| AHD05678.1 | nonribosomal\_peptide\_ligase\_subunit | BGC0000402 | Paenilarvins\_biosynthetic\_gene\_cluster | 29 | 46.1835003855 | 194 | 8.9e-49 |
| AGZ03650.1 | SevA | BGC0000426 | Sevadicin\_biosynthetic\_gene\_cluster | 27 | 47.5713184271 | 193 | 1.2e-48 |
| AAX31557.1 | peptide\_synthetase\_1 | BGC0000336 | Daptomycin\_biosynthetic\_gene\_cluster | 30 | 47.3400154202 | 193 | 1.2e-48 |
| CAJ21198.2 | non-ribosomal\_peptide\_synthetase | BGC0000297 | Aeruginoside\_biosynthetic\_gene\_cluster | 29 | 47.6484194295 | 193 | 1.2e-48 |
| AIW82277.1 | PuwA | BGC0001125 | Puwainaphycins\_biosynthetic\_gene\_cluster | 33 | 35.0809560524 | 193 | 1.5e-48 |
| ACG60778.1 | NRPS(A/PCP) | BGC0001058 | Zorbamycin\_biosynthetic\_gene\_cluster | 31 | 46.800308404 | 192 | 2.6e-48 |
| CUI25745.1 | Nonribosomal\_peptide\_synthase | BGC0001354 | U-68204\_biosynthetic\_gene\_cluster | 29 | 47.1858134156 | 192 | 2.6e-48 |
| BAO98812.1 | putative\_nonribosomal\_peptide\_synthetase | BGC0001002 | Jawsamycin\_biosynthetic\_gene\_cluster | 29 | 48.1881264456 | 191 | 4.4e-48 |
| AAN32981.1 | BarG | BGC0000962 | Barbamide\_biosynthetic\_gene\_cluster | 30 | 35.8519660756 | 191 | 5.8e-48 |
| ABC36203.1 | thiotemplate\_mechanism\_natural\_product\_synthetase | BGC0001102 | Malleilactone\_biosynthetic\_gene\_cluster | 31 | 36.6229760987 | 191 | 7.6e-48 |
| EFL02193.1 | amino\_acid\_adenylation\_domain-containing\_protein | BGC0000996 | Frontalamides\_biosynthetic\_gene\_cluster | 33 | 38.5505011565 | 191 | 7.6e-48 |
| CBJ90358.1 | putative\_Peptide\_synthetase | BGC0000465 | Xenortide\_biosynthetic\_gene\_cluster | 31 | 34.9267540478 | 191 | 7.6e-48 |
| CUI25672.1 | Thiolactomycin\_Hybrid\_PKS-NRPS\_system | BGC0001353 | Thiolactomycin\_biosynthetic\_gene\_cluster | 29 | 46.1835003855 | 190 | 1.7e-47 |
| AGD80616.1 | amino\_acid\_adenylation\_domain-containing\_protein | BGC0000394 | Naphthyridinomycin\_biosynthetic\_gene\_cluster | 32 | 39.9383191981 | 189 | 2.9e-47 |
| AAG29780.1 | acyl-CoA\_synthetase | BGC0000833 | Coumermycin\_A1\_biosynthetic\_gene\_cluster | 28 | 47.3400154202 | 187 | 8.4e-47 |
| AAN65224.1 | peptide\_synthetase-like\_protein | BGC0000832 | Clorobiocin\_biosynthetic\_gene\_cluster | 28 | 47.2629144179 | 187 | 8.4e-47 |
| ACJ04424.1 | aureobasidin\_A1\_biosynthesis\_complex | BGC0000307 | Aureobasidin\_A1\_biosynthetic\_gene\_cluster | 29 | 48.1881264456 | 186 | 1.4e-46 |
| WP\_010369430.1 | Non-ribosomal\_peptide\_synthase | BGC0000314 | Bromoalterochromides\_biosynthetic\_gene\_cluster | 26 | 48.8049344641 | 186 | 1.4e-46 |
| ACN39014.1 | putative\_nonribosomal\_peptide\_synthetase\_TomA | BGC0000448 | Tomaymycin\_biosynthetic\_gene\_cluster | 29 | 47.3400154202 | 186 | 1.9e-46 |
| CAD29794.1 | peptide\_synthetase | BGC0001015 | Microcystin\_biosynthetic\_gene\_cluster | 26 | 48.265227448 | 183 | 1.6e-45 |
| AHA12079.1 | polyketide\_synthase\_type\_1 | BGC0001172 | Chlorizidine\_A\_biosynthetic\_gene\_cluster | 30 | 37.2397841172 | 183 | 2.1e-45 |
| BAP16693.1 | nonribosomal\_peptide\_synthetase | BGC0000376 | JBIR-34,\_JBIR-35\_biosynthetic\_gene\_cluster | 28 | 49.4217424827 | 181 | 4.6e-45 |
| ABC35796.1 | polyketide\_synthase,\_putative | BGC0001102 | Malleilactone\_biosynthetic\_gene\_cluster | 32 | 31.4572089437 | 181 | 4.6e-45 |
| ALK27916.1 | non-ribosomal\_peptide\_synthase | BGC0001233 | Feglymycin\_biosynthetic\_gene\_cluster | 28 | 48.5736314572 | 181 | 7.8e-45 |
| ALJ49920.1 | TtmI | BGC0001236 | Thiotetroamide\_biosynthetic\_gene\_cluster | 29 | 45.7979953739 | 181 | 7.8e-45 |
| ADZ45339.1 | non-ribosomal\_peptide\_synthetase | BGC0001020 | Muraymycin\_biosynthetic\_gene\_cluster | 32 | 39.2444101773 | 181 | 7.8e-45 |
| CBL93716.1 | NRPS\_A-domain | BGC0000360 | GE81112\_biosynthetic\_gene\_cluster | 31 | 39.9383191981 | 180 | 1.3e-44 |
| CUI25728.1 | Nonribosomal\_peptide\_synthase | BGC0001355 | U-68204\_biosynthetic\_gene\_cluster | 28 | 49.8072474942 | 180 | 1.7e-44 |
| AAF67501.2 | NovH | BGC0000834 | Novobiocin\_biosynthetic\_gene\_cluster | 28 | 47.7255204318 | 178 | 3.9e-44 |
| CCP45167.1 | Peptide\_synthetase\_MbtF\_(peptide\_synthase) | BGC0001021 | Mycobactin\_biosynthetic\_gene\_cluster | 31 | 40.0154202005 | 178 | 6.6e-44 |
| EYT83459.1 | hypothetical\_protein | BGC0001213 | Nataxazole\_biosynthetic\_gene\_cluster | 34 | 29.1441788743 | 176 | 1.9e-43 |
| AEC14348.1 | nonribosomal\_peptide\_synthetase | BGC0000377 | Koranimine\_biosynthetic\_gene\_cluster | 28 | 43.8704703161 | 176 | 1.9e-43 |
| ABM21571.1 | CrpC | BGC0000975 | Cryptophycin\_biosynthetic\_gene\_cluster | 30 | 34.3870470316 | 176 | 2.5e-43 |
| CBW45640.1 | PII\_peptide\_synthetase | BGC0000952 | Pristinamycin\_biosynthetic\_gene\_cluster | 28 | 52.7370855821 | 175 | 4.3e-43 |
| BAH33409.1 | putative\_non-ribosomal\_peptide\_synthetase | BGC0000371 | Heterobactin\_biosynthetic\_gene\_cluster | 29 | 46.6461063994 | 172 | 2.8e-42 |
| CCA29203.1 | non-ribosomal\_peptide\_synthetase/polyketide\_synthase | BGC0000955 | Althiomycin\_biosynthetic\_gene\_cluster | 28 | 49.1904394757 | 171 | 4.8e-42 |
| CAQ18839.1 | hybrid\_polyketide\_synthase/nonribosomal\_polypetide\_synthetase | BGC0000954 | Ajudazol\_biosynthetic\_gene\_cluster | 32 | 35.0809560524 | 171 | 8.1e-42 |
| WP\_010369428.1 | phenylalanine\_racemase | BGC0000314 | Bromoalterochromides\_biosynthetic\_gene\_cluster | 28 | 46.6461063994 | 169 | 2.4e-41 |
| AAR87759.1 | ZmaJ | BGC0001059 | Zwittermycin\_A\_biosynthetic\_gene\_cluster | 27 | 40.4780262143 | 169 | 3.1e-41 |
| AFD30953.1 | CrmB | BGC0000966 | Caerulomycin\_A\_biosynthetic\_gene\_cluster | 29 | 46.8774094063 | 168 | 5.3e-41 |
| ACA97580.1 | PmxE | BGC0000408 | Polymyxin\_biosynthetic\_gene\_cluster | 30 | 37.6252891288 | 167 | 1.2e-40 |
| ctg1\_orf003 |  | BGC0000334 | Cyclosporin\_biosynthetic\_gene\_cluster | 25 | 53.5851966076 | 165 | 5.8e-40 |
| CUI25701.1 | amino\_acid\_adenylation\_domain-containing\_protein\_potentially\_involved\_in\_thiotetronic\_acid\_synthesis | BGC0001352 | Thiotetronate\_Tu\_3010\_biosynthetic\_gene\_cluster | 27 | 45.7979953739 | 164 | 7.6e-40 |
| AEC14346.1 | nonribosomal\_peptide\_synthetase | BGC0000377 | Koranimine\_biosynthetic\_gene\_cluster | 25 | 48.033924441 | 164 | 7.6e-40 |
| ADJ63842.1 | non-ribosomal\_peptide\_synthetase\_protein | BGC0000424 | Serobactins\_biosynthetic\_gene\_cluster | 28 | 47.2629144179 | 164 | 9.9e-40 |
| ADZ45333.1 | NRPS/PKS\_hybrid | BGC0001020 | Muraymycin\_biosynthetic\_gene\_cluster | 29 | 46.800308404 | 163 | 2.2e-39 |
| AJM89738.1 | PmxE | BGC0001192 | Colistin\_biosynthetic\_gene\_cluster | 30 | 35.3893600617 | 163 | 2.2e-39 |
| AEZ51520.1 | PmxE | BGC0001153 | Polymyxin\_biosynthetic\_gene\_cluster | 29 | 41.1719352352 | 160 | 1.4e-38 |
| AAS92545.1 | SirP | BGC0001044 | Sirodesmin\_biosynthetic\_gene\_cluster | 28 | 46.4148033924 | 160 | 1.4e-38 |
| AKP45399.1 | CysK | BGC0001413 | Cystobactamide\_biosynthetic\_gene\_cluster | 28 | 53.1225905937 | 158 | 4.2e-38 |
| AEC14347.1 | nonribosomal\_peptide\_synthetase | BGC0000377 | Koranimine\_biosynthetic\_gene\_cluster | 26 | 46.9545104086 | 156 | 2.1e-37 |
| ABC34483.1 | nonribosomal\_peptide\_synthetase,\_putative | BGC0000961 | Bactobolin\_biosynthetic\_gene\_cluster | 27 | 48.7278334618 | 149 | 1.9e-35 |
| AGC83576.1 | NRPS | BGC0000818 | Notoamide\_biosynthetic\_gene\_cluster | 28 | 50.5782575173 | 145 | 4.8e-34 |
| ALU98443.1 | hypothetical\_protein | BGC0001397 | Lidamycin\_biosynthetic\_gene\_cluster | 29 | 39.8612181958 | 143 | 1.4e-33 |
| ABR67749.1 | CmnF | BGC0000316 | Capreomycin\_biosynthetic\_gene\_cluster | 29 | 36.1603700848 | 142 | 4e-33 |
| AAL06681.1 | A-domain\_type\_II\_peptide\_synthetase | BGC0000965 | C-1027\_biosynthetic\_gene\_cluster | 29 | 40.1696222051 | 141 | 9e-33 |
| AGQ43600.1 | HC-toxin\_synthetase | BGC0001166 | HC-toxin\_biosynthetic\_gene\_cluster | 27 | 50.5782575173 | 140 | 1.5e-32 |
| BAH23995.1 | nonribosomal\_peptide\_synthetase | BGC0000356 | Fumitremorgin\_biosynthetic\_gene\_cluster | 26 | 46.1835003855 | 139 | 2.6e-32 |
| AFO59871.1 | ChxJ | BGC0000175 | Cycloheximide\_/\_actiphenol\_biosynthetic\_gene\_cluster | 31 | 29.3754818813 | 134 | 8.4e-31 |
| AAA33023.2 | HC-toxin\_synthetase | BGC0000370 | HC-toxin\_biosynthetic\_gene\_cluster | 25 | 48.1110254433 | 133 | 2.4e-30 |
| AAS98783.1 | JamL | BGC0001001 | Jamaicamide\_biosynthetic\_gene\_cluster | 30 | 22.66769468 | 129 | 2.7e-29 |
| EPE34341.1 | Acetyl-CoA\_synthetase-like\_protein | BGC0001035 | Pneumocandin\_biosynthetic\_gene\_cluster | 25 | 47.9568234387 | 129 | 3.5e-29 |
| CAP96445.1 |  | BGC0000420 | Roquefortine\_C\_/\_meleagrin\_biosynthetic\_gene\_cluster | 26 | 45.0269853508 | 129 | 3.5e-29 |
| AAC83656.1 | dihydroaeruginoic\_acid\_synthetase | BGC0000412 | Pyochelin\_biosynthetic\_gene\_cluster | 28 | 33.0763299923 | 124 | 6.7e-28 |
| ERG36783.1 | phenylalanine\_racemase | BGC0000299 | Alterochromides\_biosynthetic\_gene\_cluster | 30 | 31.3030069391 | 124 | 1.1e-27 |
| CAP93139.1 |  | BGC0000357 | Fungisporin\_biosynthetic\_gene\_cluster | 25 | 52.1202775636 | 120 | 1.6e-26 |
| AGY30675.1 | Ann3 | BGC0001298 | 4-Z-annimycin\_biosynthetic\_gene\_cluster | 27 | 39.9383191981 | 87 | 9e-17 |
| ADC79614.1 | BafY | BGC0000028 | Bafilomycin\_biosynthetic\_gene\_cluster | 27 | 24.3639167309 | 78 | 5.5e-14 |
| ADN13832.1 | Beta-ketoacyl\_synthase | BGC0001164 | 1-heptadecene\_biosynthetic\_gene\_cluster | 24 | 34.7725520432 | 77 | 9.3e-14 |
| ACA99172.1 | Polyketide\_synthase | BGC0001160 | Nonadecene\_biosynthetic\_gene\_cluster | 25 | 39.3215111796 | 76 | 2.7e-13 |
| ABJ90147.1 | MoeB4 | BGC0000805 | Moenomycin\_biosynthetic\_gene\_cluster | 23 | 38.3962991519 | 69 | 4.3e-11 |
| CBD77754.1 | hypothetical\_protein | BGC0000974 | Crocacin\_biosynthetic\_gene\_cluster | 26 | 27.0624518119 | 64 | 1.1e-09 |
| CBA11557.1 | non-ribosomal\_peptide\_synthetase | BGC0001046 | Streptolydigin\_biosynthetic\_gene\_cluster | 27 | 22.66769468 | 60 | 1.5e-08 |
